# Formulation, *in-vitro* characterization and clinical evaluation of curcumin *in-situ* gel for treatment of periodontitis

**DOI:** 10.1080/10717544.2016.1233591

**Published:** 2017-02-03

**Authors:** Maha M. A. Nasra, Heba M. Khiri, Heba A. Hazzah, Ossama Y. Abdallah

**Affiliations:** 1 Department of Pharmaceutics, Faculty of Pharmacy, Alexandria University, Alexandria, Egypt and; 2 Department of Pharmaceutics, Faculty of Pharmacy and Drug Manufacturing, Pharos university in Alexandria, Alexandria, Egypt

**Keywords:** Chemical stability, curcumin, *in-situ* gel, mucoadhesive, periodontitis

## Abstract

This study aimed to develop syringeable *in-situ* curcumin (cur) gel for the treatment of periodontal pockets as well as to evaluate the clinical efficacy of Cur *in-situ* gel formulation. Different *in-situ* gel formulations of Cur were prepared using 30% of pluronic F127, and 1% of carbopol P934. The formulations were evaluated regarding gelation temperature, pH, viscosity, syringeability study, *in-vitro* release and chemical stability of cur. The effect of aging of gel formulations for 3months in refrigerator was investigated. The selected formulation was clinically evaluated through the determination of probing depth, plaque index, and bleeding index at baseline and 1 month after application. The formulations showed accepted gelation temperature ranging from 28 to 34 °C and all had pH value of 4. The viscosity of the formulations at 4 °C ranged from 19 000 to 37 000 cP. All formulations were easily syringeable through 21 gauge needle at cold temperature. Curcumin stability during the release study was maintained. Aging showed no significant effect on release profile, drug content, or the pH after 3 months, while it showed a slight increase in viscosity with concomitant decrease in gelation temperature. Selected formulations delivered into periodontal pocket evaluated clinically showed to be effective. The treated group revealed that the adjunctive use of intracrevicular 2% curcumin *in-situ* gel adjunct to mechanical treatment in patients with adult periodontitis could aid in significant clinical reduction of probing depth, bleeding index, and to less extent of plaque. This indicates that curcumin in this novel drug delivery system is an excellent candidate for periodontal disease treatment.

## Introduction

Periodontal diseases are highly prevalent and can affect up to 90% of the worldwide population. Gingivitis, the mildest form of periodontal disease, is classified as a nondestructive periodontal disease, caused by the bacterial biofilm (dental plaque) that accumulates on teeth adjacent to the gingiva (gums). Some of the most common symptoms associated with gingivitis are bad breath, and bleeding, bright, tender or swollen gums.

Although gingivitis could be reversible, if left untreated, it can progress to a form of periodontal disease that can be highly destructive, which could result in loss of connective tissue and bone support causing tooth loss in adults (Pihlstrom et al., 2005). Periodontitis can be linked to an increased risk of infective endocarditis, cardiovascular disease, stroke diabetes mellitus, respiratory disease and adverse pregnancy outcomes.

Management of periodontal diseases usually involves various modalities, particularly the use of systemic antimicrobial and anti-inflammatory agents (Pihlstrom et al., 2005; Babu et al., 2011). However, long-term use of systemic drugs is fraught with potential danger including resistant strains and superimposed infections (Garala et al., 2013). The increasing resistance in many common pathogens to currently used therapeutic agents raised the interest to discover novel anti-infective natural compound derived from plants origin. Curcumin, a polyphenolic compound which has been used as herbal medicine and food for nearly 4000 years has been given a great attention. Curcumin shows anti-inflammatory, antioxidant, antimicrobial, immunostimulant, antiseptic, and antimutagenic properties. In addition to pain suppression and wound healing, it is relatively nontoxic and safe. Due to all these properties, it is proposed to be advantageous in dentistry as well (Nagpal & Sood, 2013).

Curcumin acts as anti-inflammatory agent by reducing inflammatory mediators involved in periodontitis. It also enhances wound healing by causing an increase in fibronectin and transforming growth factor â transcription (Guimarães et al., 2011; Rastogi et al., 2012). Worth mentioning is that Curcumin has an antiadhesion effect against *Streptococcus* mutans on extracellular matrices and tooth surfaces, which are crucial receptors for dental caries and infective endocarditis (Song et al., 2012). However, its poor oral bioavailability, aqueous solubility, and permeability, in addition to its rapid metabolic disposition and hydrolytic degradation in neutral and alkaline media hindered clinical application.

Local delivery of curcumin targeting oral cavity introduces the potential to maximize its local effect by avoiding its extensive intestinal and first pass metabolism, and prolonging drug residence at the site of action. Thus, introducing curcumin locally in a system maintaining its chemical stability and improving its solubility provides a very promising approach for the treatment of periodontitis.

*In-situ* gel-forming formulations is currently a novel idea to deliver drugs as a liquid dosage form, which then form strong gels after application at delivery site, thus prolonging the residence time of the active substance (Garala et al., 2013). Amongst *in-situ* gelling polymers, thermosensitive systems containing poloxamer have been investigated as a convenient dosage form for application into periodontal pocket. Furthermore, semisolid formulations consisting of mucoadhesive polymers such as polycarbophil, hydroxypropyl cellulose, polyvinylpyrrolidone, and carbopol have been proposed in order to improve the intimacy of contact and also increase the residence time of the dosage form in the periodontal pocket.

The present investigation aimed to design and evaluate curcumin *in-situ* gel formulations combining a thermosensitive polymer “poloxamer” as well as mucoadhesive one, namely “carbopol” as a local drug delivery within pockets for the treatment of periodontitis. The prepared formulations are aimed to stabilize curcumin to guarantee its efficacy through the whole time of application. Those formulations combines the advantages of the ease of administration, reduced frequency of administration, and providing a sustained drug release in order to increase clinical efficacy and patient compliance.

## Materials and methods

Curcumin Cur (Hebei Food Additive Co., Ltd, China) (purity 95%), polyethylene glycol (PEG) 400, potassium di-hydrogen phosphate and sodium lauryl sulfate (SLS) (El-Nasr Pharmaceutical Co., Egypt) and tri-ethanol amine (TEA) (Nice Chemicals, Pvt Ltd, Kochi, India), Pluronic 127 (Kolliphore 407) (Kind Gift from BASF, Ludwigshafen, Germany), PEG 7-glyceryl-cocoate (Galaxy, Navi, Mumbai, India), Carbopol P 934 (a sample gift, Lubrizol, Belgium.)

### Solubility studies

Solubility of curcumin in PEG 400 or propylene glycol (PG) was determined by adding an excess amount of curcumin to glass vials containing 5 ml of each solvent. Vials were shaken in a thermostatically controlled water bath at 37 °C until equilibrium. The supernatants were filtered, diluted properly with acetone and assayed using UV-spectrophotometer at 421 nm for curcumin. The concentration was calculated on a previously constructed calibration curve of curcumin solutions of successive dilution in acetone.

### Preparation of curcumin *in-situ* gel

Carbopol–poloxamer gel of curcumin was prepared by the cold method (Schmolka, 1972). Carbopol P934 (1% w/v) was initially dissolved in deionized water using a magnetic stirrer. Following complete dissolution, the solution was cooled in an ice bath, pluronic F127 (30% w/v) was then added slowly with continuous stirring. The mixture was transferred to fridge at 4^о^C for 24 h to ensure complete wetting and removal of entrapped air bubbles. Curcumin powder was slowly added to the prepared polymers solution making 1%, and or 2% w/w or curcumin previously dissolved in an appropriate amount of PEG 400 (140 mg/ml) or ethanol (100 mg/ml) was then added to polymer solution with stirring in ice bath. All samples were then transferred into amber bottles and stored in refrigerator. For *in-situ* gel formulation containing curcumin dissolved in ethanol, formulation was left on stirrer over night to allow ethanol evaporation before storage in refrigerator.

### *In-vitro* evaluation of curcumin *in-situ* gel

#### Gelation temperature

The gelation temperature was determined using the test-tube-inverting method. A volume of 2 ml of the *in-situ* gel was placed in a test tube, which was then immersed in a water bath at 15 °C. The temperature of the water bath was then gradually increased, samples were examined every 2 minutes, and the gelation temperature was recorded when the gel stops flowing upon test tube inversion at 90°. The readings were taken for an average of 3 samples (Vadnere et al., 1984; Gilbert et al., 1987).

#### Determination of pH

The pH of the gel was determined using a calibrated pH meter at 4 °C. The readings were taken for an average of 3 samples.

#### Viscosity measurement

Viscosity of sols was measured using Brookfield viscometer (model DVII, Engineering Laboratories, Middleboro, MA) spindle no 01 at 20 r.p.m. at temperature 4 °C and 37 °C. The experiment was carried out in triplicate.

#### Syringeability study

The ability of the prepared formulations to flow easily through a syringe of 21 gauge needle was assessed using the same method employed by Maheshwari et al. (2006). One ml of the cold gel was filled in 21 gauge needle syringe and the ability of the gel to flow under normal handling pressure was assessed.

#### Chemical stability of curcumin

Chemical stability was evaluated at different concentrations of pluronic F127. Stock solution of curcumin in acetone at a concentration of 500 μg/ml was prepared and diluted to 10 μg/ml using 0.02%, 0.05% and 10% of pluronic F127 in PBS pH 6.8. The samples were filtered through 0.45 μm pore-sized filter and filtrates were then stored at 37 °C in a thermostatically controlled water bath. Samples were withdrawn at predetermined time intervals and analyzed using an UV spectrophotometer for curcumin at *λ*_max_ 421 nm.

### *In-vitro* release studies

A sample of 1 ml of 1% curcumin gel or 0.5 ml of 2% gel (equivalent to 10 mg curcumin) were placed into a dialysis membrane 7 cm long (Visking, cutoff 12–14 kD, SERVA, Germany). Bags were then suspended in 50 ml of (ethanol: water 1:1) preheated at 37 ± 0.5 °C in shaking water bath at 37 °C and 25 strokes per min. At predetermined time intervals, one milliliter sample was withdrawn and replaced with an equal volume of fresh medium. The whole release media were changed and replaced with fresh media every day (24 h) during the release studies duration (up to one week). Samples were diluted and analyzed using an UV spectrophotometer for curcumin concentration at *λ*_max_ 431 nm. The cumulative amount of drug released was calculated based on a calibration curve of curcumin in ethanol to water (1:1). All experiments were done in triplicate. Formulations tested were un-neutralized at pH4, and neutralized with triethanolamine (TEA) to pH 6.8 immediately prior to its placement into the dialysis bag.

### Release kinetics

The mechanism of drug release from the selected formulation was investigated using the mathematical models: zero-order kinetics, first-order, Higuchi square root of time and Peppas using DD-solver excel sheet software application. The Peppas model has been widely used in many studies for the analysis of the drug release kinetics in matrix systems. For the Fickian diffusion, *n* = 0.5 while for the anomalous transport, *n* is between 0.5 and 1 and for a case II transport (zero-order release), *n* = 1 (Siepmann & Siepmann, 2008).

### Effect of aging

The effect of storage on selected formulation over three months of storage in a refrigerator was investigated. Samples were stored in a clear well closed amber glass bottles and at predetermined time intervals, samples were evaluated for any change in the *in-vitro* release, viscosity, pH, gelation temperature as well as drug content.

### Statistical analysis

The *in-vitro* release profile at 0 time, after 1 month and after 3 months were compared using the Wilcoxon signed ranks test. A value of *p* < 0.05 was considered statistically significant.

### Clinical evaluation of curcumin *in-situ* gel for treatment of periodontitis

#### Study population

Twenty systemically healthy patients aged 35–55 years of both sexes who had been diagnosed with adult (moderate to advanced chronic) periodontitis with isolated localized periodontal pocket of measuring ≥5 mm in addition to marks of bleeding on probing were selected for the study. The patients were receiving outpatient treatment in the Department of Periodontology, 6th October University in Cairo.

Patients were divided into two groups: After signing an informed consent, eligible patients were randomly assigned to two groups, Group I (*n* = 10) and Group II (*n* = 10). The work described has been carried out in accordance with The Code of Ethics of the World Medical Association (Declaration of Helsinki) for experiments involving humans (Association. WM, 2001).

Control group (I): received scaling and root planing only.

Treated group (II): in which, on completion of scaling and root planing, pockets were completely dried using an air syringe and the pockets were isolated with cotton rolls to prevent contamination from saliva. The gel formulation (G_2_) stored at 4 °C (before application) was carried in a 1 ml syringe with a needle attached to it. The liquid was placed in the periodontal pocket until overfilling was obtained and as the temperature increased, gel formation occurred. Gentle force was applied so that the material filled the depths of the pocket. The application was repeated once weekly over three weeks period.

Patients were instructed not to brush the area of application, not to chew hard or sticky food, not to floss on the treated site, and not to probe the area with the tongue, a finger or a tooth pick and not to take any antibiotic, or anti-inflammatory drugs. Patients were also told to report immediately discomfort, burning sensation or any other allergic reaction if any. Subjects were recalled one month after the first placement of the local experimental drug to measure clinical parameters.

#### Clinical variables studied

The following clinical indices were recorded before starting the scaling and root planning (baseline) and one month post baseline. For each patient, the following clinical parameters were recorded on 6 points per tooth mesio-mid and disto-buccal, mesio-mid and disto-lingual:

1. Periodontal probing depth (PD) as described by Glavind & Löe (1967).
2. Bleeding index: Checchi et al. (2009) A standard periodontal probe was used to measure the gingival bleeding and results were categorized as follows: (0 = No bleeding within 30 s of probing, 1 = Bleeding within a few seconds of probing, 2 = Immediate bleeding on probing, 3 = Bleeding along gingival sulcus with slightest touch)
3. Plaque index (PI): The amount of dental plaque was scored according to the plaque index of Silness and Löe (Löe & Silness, 1963; Silness & Löe, 1964).

The scores from 0 to 3 are as follows:

Score 0 = Gingival areas of tooth free from plaque, Score 1 = No plaque was observed *in-situ* by the unaided eye, but plaque is made visible on the tip of a probe after it has been moved over the tooth surface at the entrance of the gingival crevice, Score 2 = Gingival area covered by a thin to moderately thick layer of plaque visible to the naked eye. Score 3 = Heavy accumulation of soft matter, the thickness of which fills the crevice produced by the gingival margin and the tooth surface.

#### Statistical analysis of the data

Data were analyzed using IBM SPSS software package version 20.0 (Kirkpatrick & Feeney, 2012). Quantitative data were described using mean and standard deviation, median, minimum and maximum. The distributions of quantitative variables were tested for normality using the Kolmogorov–Smirnov test, Shapiro–Wilk test and D'Agstino test, also Histogram and QQ plot were used for vision test. If it reveals normal data distribution, parametric tests was applied. If the data were abnormally distributed, non-parametric tests were used. For normally distributed data, comparison between two independent population were done using the independent *t*-test, also paired *t*-test was used to analyze two paired data, comparison between different periods using ANOVA with repeated measures and *post-hoc* test was assessed using Bonferroni adjusted. For abnormally distributed data, comparison between two independent populations was done using the Mann–Whitney test. To compare between the different periods, Friedman test as well as the Wilcoxon signed ranks test were applied. Significance test results are quoted as two-tailed probabilities. Significance of the obtained results was judged at the 5% level.

## Results and discussion

In the present study, an attempt was made to develop and evaluate a combination of thermosensitive and pH-sensitive *in-situ* gel formulations of curcumin having controlled release for direct placement into periodontal pocket. In this study, pluronic F127 has been used as the thermoreversible polymer while carbopol P934 has been used as pH-sensitive and mucoadhesive one.

### Solubility study

Solubility of curcumin in PEG400 and PG were carried out to select an appropriate solubilizing agent for curcumin to be introduced into some formulations. Results revealed that solubility of curcumin in PEG400 was 140 mg/ml compared and 5.35 mg/ml for PG. These results were in agreement with Zhongfa et al. who reported that PEG400 was capable of solubilizing curcumin to a higher extent (Zhongfa et al., 2012). W.W. Quitschke (2012) explained the solubilization power of PEG by its amphiphilic properties confirming Mandeville et al. (2009) and Sun et al. (2008) observations that curcumin requires hydrophobic and hydrophilic moieties balance, in addition they described the solubility behavior of curcumin as amphipathic.

### Preparation of curcumin insitu gel

Gel was prepared using the cold method to prevent lumps formation in the case of hot process. Formulations G_1_ and G_2_ prepared by dispersing curcumin powder in cold carbopol–poloxamer solution, upon storage in refrigerator, only partial curcumin precipitation was observed. For comparison, formulations G_3_, G_4_ and G_5_ containing solubilized curcumin were prepared using two different solvents, PEG400 and ethanol (Jagetia & Aggarwal, 2007).

### *In-vitro* evaluation of curcumin *in-situ* gel

#### Gelation temperature (Tsol–gel)

Tsol–gel of formulations are shown in Table 1. All formulations showed accepted gelation temperature; higher than room temperature and lower than body temperature (28–34 °C), except G_5._ Formulations G_1_ and G_2_ containing suspended curcumin (1% and 2%) showed higher gelation temperature (34 °C) than G_3_ and G_4_ containing curcumin dissolved in PEG400 1% and 2% showing gelation temperature (30 °C and 28 °C, respectively) in spite of using the same concentration of thermosensitive polymer (30% pluronic F127) in all formulations, this might be due to the presence of PEG400 added to solubilize curcumin. These results were in agreement with observation of Bilensoy et al. who reported that the gelation temperature of pluronic *in-situ* gel of clotrimazole decreased upon using of PEG400 as a solubilizing agent and an excess amount of PEG causes the formation of gels even at 4 °C (Bilensoy et al., 2006). Formulation G_5_ containing curcumin dissolved in ethanol did not form gel at body temperature 37 °C which was previously observed by Dumortier et al. who reported that ethanol reduce the gel strength and bioadhesive force and increase Tsol–gel (Dumortier et al., 2006).

**Table 1.** *In-vitro* evaluation of curcumin *in-situ* gel formulations containing 30% pluronic F127 and 1% carbopol P934, of pH 4 at zero time and after three months storage in refrigerator.

		*T*_sol–gel_		Viscosity (cP) at 4°C	
Code	Cur form, concentration	Zero time	After 3 months	Zero time	After 3 months
G1	Dispersed, 1%	34 °C	32 °C	19 000 ± 250	21 000 ± 120
G2	Dispersed, 2%	34 °C	32 °C	19 000 ± 200	21 500 ± 180
G3	Dissolved in PEG400, 1%	30 °C	20 °C	30 000 ± 300	42 000 ± 250
G4	Dissolved in PEG400, 2%	28 °C	–	37 000 ± 400	–
G5	Dissolved in ethanol, 1%	> 37 °C		–	

#### pH measurement

The pH value for *in-situ* gel formulations was found to be 4. This low pH maintains carbopol P934 in sol form before administration. It is also important for maintaining curcumin chemical stability. It is worth noting that upon placement into the periodontal pockets, it would be in contact with physiological pH and temperature 37 °C resulting in the formation of stiff gel with high strength and mucoadhesion properties.

#### Viscosity studies

The amount of gel that can be introduced in the periodontal pockets is extremely low, and it is necessary to raise the viscosity for better retention in the periodontal pocket and better control of drug release. On the other hand, to instill easily in the periodontal pocket, the formulation should have an optimum viscosity. Hence, the viscosity of sols of various formulations was determined at 4 °C and at 20 rpm and was used for comparative evaluation. The viscosities of all the developed gel formulations at 4 °C were in the range 19 000–36 000 cP (centipoise). Formulations G_1_ and G_2_ showed the lower viscosity 19 000 cP, while G_3_ and G_4_ showed higher viscosity 30 000 and 37 000 cP, respectively. This difference was in agreement with the differences in the gelation temperature, which is most probably due to the presence of PEG400 and its concentration (Table 1). It is worth mentioning that the determination of viscosity at 37 °C was not feasible due to that the stiff gel formed

#### Syringeability study

All the developed formulations were easily syringeable through 21-gauge needle at cold temperature.

### Chemical stability of curcumin

Chemical stability of curcumin at the application site should be considered to ensure its clinical efficacy. Curcumin stability depended on the amount of pluronic F127 present in the applied dose, the available volume of fluid for drug release and the residence time of the formulation at the application site. When cold liquid *in-situ* gel is injected into the periodontal pockets (after mechanical treatment), it will be converted to gel and then it will be exposed to inflammatory exudates and to the crevicular fluid, resulting in the formation of diffusion gel layer through which the drug will diffuse.

Experimental design to investigate curcumin stability in the applied gel, in the diffusion gel layer and also the curcumin released in crevicular fluid might be difficult so theoretical calculations were assumed to be helpful to predict the curcumin stability upon application. The stability of curcumin in the applied gel and the diffusion layer is governed by a high concentration of F127, so stability study was conducted in 10% F127 not only to be in solution state at 37 °C during the study, but also because F127 concentration decrease by time.

**Figure 1.**
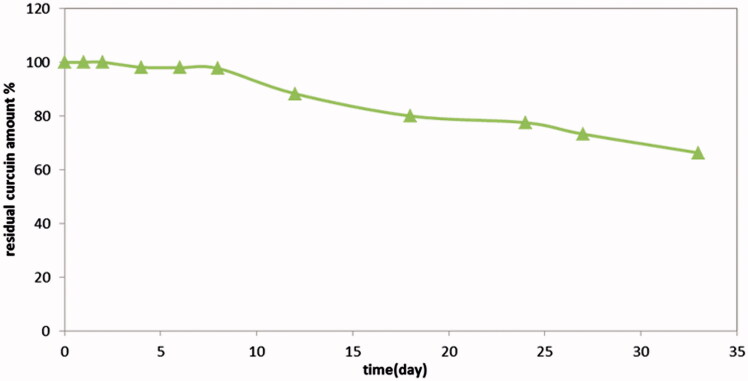
Chemical stability of curcumin in 10% pluronic F127 conc in PBS pH 6.8.

Results revealed that not less than 97% of drug remained intact after 8 days in 10% pluronic F127 aqueous solution at 37 °C, while the expected release period was one week, illustrated in Figure 1.

On the other hand, stability of curcumin released in exudates is governed by a small concentration of pluronic F127 present in the small dose applied. The common applied intrapocket dose was in μl due to pocket small size, and this dose was supposed to remain in the pocket up to one week (as expected from the release study). The periodontal clearance was reported to be 40 times per hour. The resting fluid volume in a 5 mm periodontal pocket deep is 0.5 μl that means, 0.5 μl/1.5 min and 20 μl/h. Thus, by suggesting that the applied dose was about 50–200 μl gel once weekly, upon *in-situ* gel application in pocket, the expected pluronic F127 concentration available over an hour would be approximately 0.02–0.075 g.

**Figure 2.**
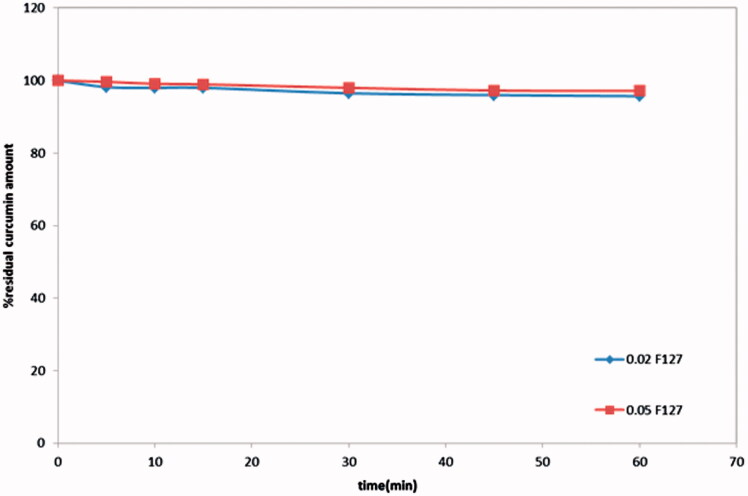
Chemical stability of curcumin in 0.02% and 0.05% pluronic F127.

The stability studies were conducted at the lower expected F127 concentrations in PBS pH 6.8 (Figure 2), results revealed that concentrations of pluronic F127 as low as 0.02% and 0.05% in PBS pH 6.8 at 37 °C was able to maintain the stability of curcumin, where not less than 95.7% and 97% of drug content remained intact after 1 hour, respectively.

Based on these findings, it was suggested that curcumin *in-situ* gel formulations containing 30% pluronic F127 is assumed to be capable of delivering curcumin into the periodontal pockets and maintaining its stability within the gel and diffusion layer as well as at the application site after drug is released.

### *In-vitro* release study

#### Selection of the release media

*In-vitro* release profiles give important information on the efficiency of the delivery system proposed for controlling drug release. In the current study, the dialysis technique was chosen as it could reproduce the situation of a formulation applied into the periodontal pocket; in this case, the carrier is presumably surrounded by a stagnant layer, causing slow diffusion of the drug.

Different release media were tested to select an appropriate one such as 0.5%, 1% SLS in PBS (pH 6.8), 0.5%, 1% SLS in distilled water, 30% PEG in PBS (pH 6.8), 30% PEG in distilled water, and different ethanol water mixtures with different proportions (20, 30, 40 and 50%).

Amongst all the solutions tested, ethanol:water (1:1) showed to be the best choice, as 100% of drug was released from dialysis bag within 8 h. This was attributed to the curcumin poor solubility, whereas a strong release media was required to create a high driving force, thus insuring curcumin release through the dialysis membrane.

#### Stability study of the curcumin during the release study

Chemical stability of curcumin was examined in the selected release medium (ethanol:water 1:1) and in 10% pluronic F127 in PBS (pH 6.8) to mimic condition inside the dialysis bag following the same aforementioned method for curcumin stability. Samples were withdrawn at predetermined time intervals (1, 2, 3, 4, 5, 24, 48, 72, 96, 120, 144 and 168 h) and analyzed spectrophotometrically at 431 and 421 nm, respectively.

**Figure 3.**
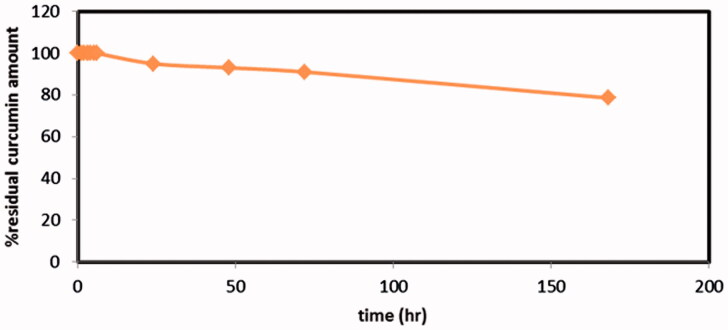
Stability profile of curcumin in the release medium (ethanol:water 1:1).

Chemical stability of Cur in the release medium and inside the dialysis bag was an important issue to design an appropriate release study. The stability study of curcumin was carried out in the selected release medium (ethanol:water 1:1). Results revealed that, 50% ethanol present in the release medium played an important role in maintaining curcumin stability whereas only limited degradation took place not more than 5%, 9% and 21% of curcumin content decomposed over 24 h, 72 h and one week, respectively (Figure 3). Therefore, this ensures the stability of released curcumin even if extended over more than 24 h, the release medium should be preferably replaced with fresh one.

Regarding chemical stability of curcumin inside the dialysis bag, pluronic F127 could not diffuse out the bag due to its high molecular weight, so the concentration used might be capable of maintaining curcumin stability during the release study even if it extends for one week.

#### *In-vitro* release studies

**Figure 4.**
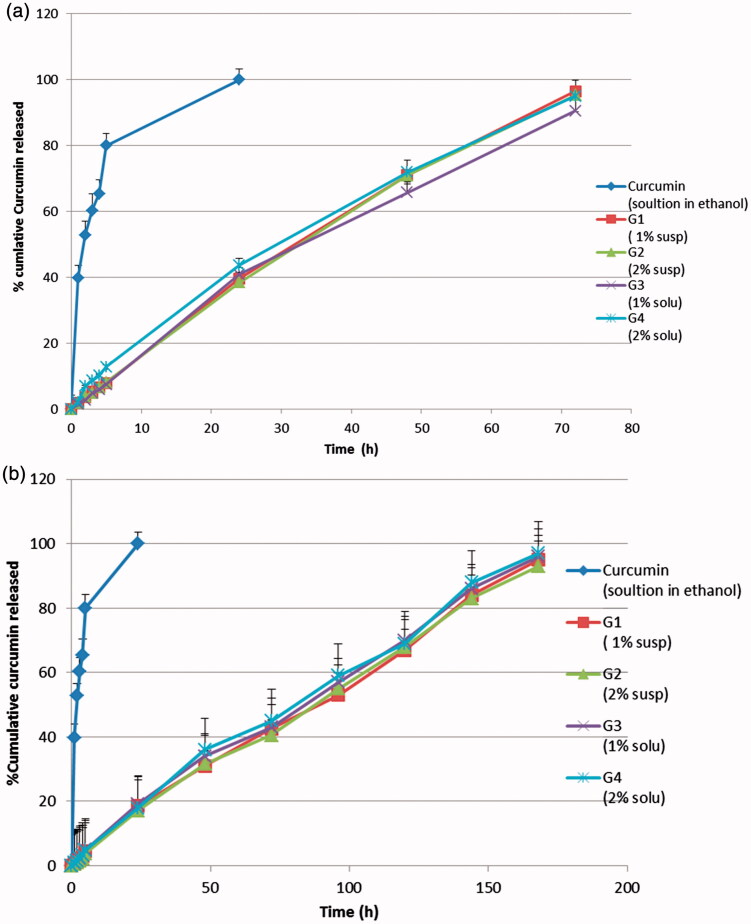
*In-vitro* release study of curcumin *in-situ* gel formulations: (a) without neutralization; (b) with neutralization.

Generally, the use of carbopol at the studied concentration 1% retarded the release of curcumin, needless to say that increasing the viscosity will increase the path of diffusional length, hence decreasing the concentration gradient and amount of drug released. Release studies were performed using the *in-situ* gel formulations with and without neutralization to pH 6.8. Formulations were neutralized immediately before the release studies to simulate the *in-vivo* condition regarding the pH and to illustrate the effect of carbopol as a pH-sensitive polymer, upon neutralization, on the release profile. The *in-vitro* release study results are shown in Figure 4. The release profiles of the four formulations without neutralization (A) were similar where 39.5, 38.4, 40.9 and 43.6% cumulative drug was released after 24 h, 71, 70.9, 65.7 and 71.9% cumulative drug was released after 48 h and 96.5, 95.3, 90.5 and 95% cumulative drug was released after 72 h from G_1_, G_2_, G_3_ and G_4_, respectively. Similarly, the release profiles of the four neutralized formulations (B) were similar where 18.5, 17.2, 19.2 and 18% cumulative drug was released after 24 h, 42.5, 40.5, 43 and 45% cumulative drug was released after 72 h, 67, 68, 70 and 69% cumulative drug was released after 120 h and 95.2, 93, 95.6, and 96% cumulative drug was released after one week from G_1_, G_2_, G_3_ and G_4_, respectively.

The similarity of drug release profiles from the four formulations was an unexpected result as the drug was incorporated in two different forms (suspended or dissolved in PEG400) and two different concentrations (1% or 2%). These results might be attributed to the fact that, in case of formulations having curcumin in dispersed form, the presence of high concentration of pluronic F127 in the diffusion layer (the rate limiting step in the release process) might be capable of *in-situ* solubilization of undissolved curcumin particles which diffuse into the diffusion layer of the gel.

On the other hand, release profiles showed a difference between non-neutralized formulations (A) and neutralized ones (B). Whereas neutralized formulations showed a more sustained drug release which extended over a week, while non-neutralized formulations extended drug release up to three days. Neutralization, the most probably reason of this difference, has activated carpobol resulting in a more viscous and strengthened gel that hindered drug release resulting in a more sustained release compared to non-neutralized ones which depended only on the gelation of pluronic polymer at 37 °C.

In this regard, the most fitting model that could describe the *in-vivo* performance may be a combination between (A) and (B) release profiles. This suggestion is based on thermal activation of the gel that may take place within seconds while neutralization may takes place gradually.

### Release kinetics

In order to investigate the drug release mechanism, the release data were fitted to different release kinetics models, zero-order, first-order and Higuchi square root of time and Peppas–Korsmeyer (Table 2). The *r*^2^ of G2 after neutralization was fitting the most with zero-order model (Peppas, 1985). Dispersion of curcumin in gel matrix showed a zero-order release with an (*r*^2^= 0.9971) and “*n*’’ value nearest to “1’’ from applying the Peppas equation came as another confirmation indicating that the drug release mechanism depended mainly on polymer swelling (Siepmann & Siepmann, 2008).

**Table 2.** Release Models for the selected formulation with and without neutralization.

	Zero order	First order	Higuchi	Korsmeyer–Peppas	
Formulation	*r^2^*	*r^2^*	*r^2^*	*r^2^*	*n*
G2 (not neutralized)	0.9929	0.9783	0.9069	0.9982	0.867
G2 (neutralized)	0.9971	0.9718	0.9229	0.9986	0.919

The release mechanism showed to follow zero order, this could be justified similarly as the explanation proposed by Siepmann and Siepmann (2008). The authors assumed that upon contact with the release medium, water diffuses into the delivery system. The mobility of polymer chain increases by increasing the water content and so does the drug molecule. Once a specific (polymer–water) concentration is reached, the macromolecular mobility steeply increases. This phenomenon is called “polymer chain relaxation” or “glassy to rubbery phase transition”. The front at which this process takes place is called “swelling front”, which separates the swollen from non-swollen matrix. This swelling front is a moving boundary. While the initial drug concentration in the delivery system exceeds drug solubility, dissolved and non-dissolved drug co-exist within the matrix. As a result of concentration gradients and the increased mobility, dissolved drug molecules diffuse out of the swollen matrix into the release medium. This guarantees that drug molecules that are released will be replaced by the dissolution of non-dissolved drug. Eventually, when all excess drugs are dissolved, the concentration within the swollen matrix decreases. The front that separates the swollen matrix containing only dissolved drug from the swollen matrix that contains both, dissolved and non-dissolved drug, is called “diffusion front” (Siepmann & Siepmann, 2008). The result of our study was in agreement with Tirnaksiz and Robinson (2005) and Hazzah et al. (2016).

### Stability testing

**Figure 5:**
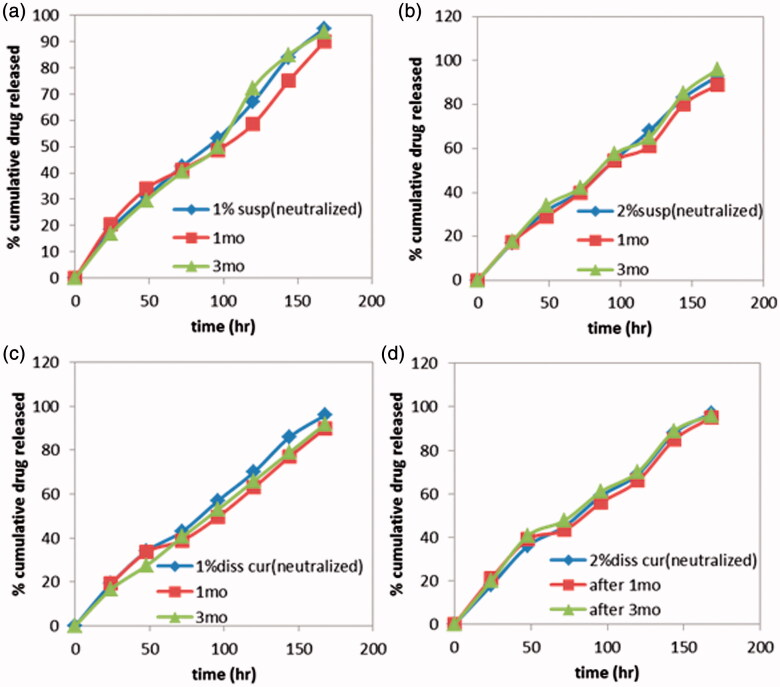
Drug release profiles of Cur *in-situ* gel formulations subjected to a stability study in refrigerator, for 3 months. (a) G1 containing 1% suspended Cur formulation, (b) G2 containing 2% suspended Cur formulation, (c) G3 containing 1% dissolved Cur formulation and (d) G4 containing 2% dissolved Cur formulation.

**Figure 6.**
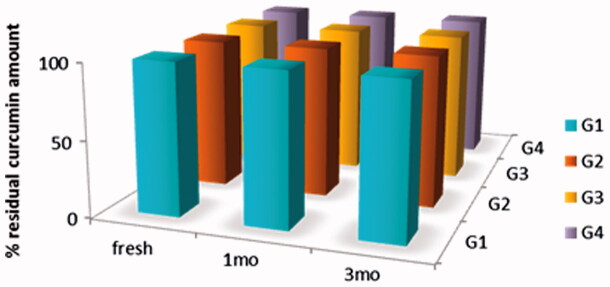
Drug content of Cur *in-situ* gel formulations subjected to a stability study in refrigerator, for 3 months.

Results revealed that storage has no significant effect on the release pattern of all formulations. (Figure 5). In addition, drug content over three months showed no change, this might be attributed to high concentration of pluronic (30%), acidic pH of formulations (pH 4) and storage temperature (Figure 6). A slight increase in viscosity was observed with concomitant decrease in gelation temperature, which was more pronounced in formulations G_3_ converted to a very viscous solution and G_4_ converted to gel in refrigerator (Table 1).

Formulation G_2_ showed most promising results regarding its physical prosperities, stability over storage period and its higher curcumin content to provide the periodontal pocket with effective dose over the drug release period. Therefore, G2 was selected for further clinical evaluation

### *In-vivo* evaluation of curcumin *in-situ* gel

In previous reports, the efficacy of curcumin to control dental diseases and inflammations has been proven after systemic or local administration (Guimarães et al., 2011; Rastogi et al., 2012). Curcumin’s low systemic bioavailability can be overcome using a local drug delivery that allows the concentration of a particular drug at the desired site. In our study, an attempt was done to evaluate the effect of 2% curcumin *in-situ* gel as adjunct to scaling and root planing (SRP) in the treatment of chronic periodontitis. In this study, it proposed to use 2% curcumin with bioadhesive *in-situ* gels as a delivery vehicle instead of mouth washes or mouth irrigants, because gels have the advantage of remaining *in-situ* longer than irrigants and the technique is simpler than manipulating strips in the periodontal pockets. Both groups were first subjected to scaling and root planning, then for experimental group II, subgingival placement of cold *in-situ* gel preparation using a simple syringe appeared to be easy, requiring only a few seconds to completely fill the periodontal pocket. The acceptability of the experimental material was observed in terms of the subject’s taste and comfort. Good biological acceptability was also seen as no evidence of burning sensation, dryness/soreness or ulcer formation

### Preliminary clinical study

#### Periodontal probing depth

Periodontal probing is one of the most widely used diagnostic tools for clinical assessment of connective tissue destruction and periodontal pocket depth in periodontal disease. On comparison between the two groups, the mean probing pocket depth reduction was significant (*p* < 0.05) at the end of one month, with better results for experimental group. Reduction in probing pocket depth in both groups might be due to the resolution of gingival inflammation after scaling and root planning and more reduction in experimental group might be due to anti-inflammatory effects of curcumin (Table 3).

**Table 3.** Comparison between the two studied groups according to pocket depth (PD), bleeding index, plaque index (PI) at base line and after one month treatment.

	PD		Bleeding index		PI	
	Baseline	1 Month	Baseline	1 Month	Baseline	1 Month
Control						
Min–Max	5.0–8.0	5.0–6.0	1.0–3.0	0.0–2.0	1.0–3.0	0.0–1.0
Mean ± SD	6.50 ± 0.97	5.50 ± 0.53	2.20 ± 0.63	1.20 ± 0.79	2.10 ± 0.74	0.70 ± 0.48
Median	7.0	5.50	2.0	1.0	2.0	1.0
Cases						
Min–Max	5.0–9.0	3.0–6.0	2.0–3.0	0.0–1.0	1.0–3.0	0.0–1.0
Mean ± SD	6.70 ± 1.25	4.40 ± 0.97	2.66 ± 0.47	0.38 ± 0.46	2.10 ± 0.74	0.40 ± 0.52
Median	7.0	4.50	3.0	0.20	2.0	0.0

#### Bleeding index

Bleeding on probing is an objective sign of inflammation. Research suggests that bleeding on probing often is the first sign of gingival inflammation. Bleeding on probing showed significant (*p* < 0.05) reduction in the two groups after one month. There was highly statistically reduction in the bleeding index in experimental group with mean bleeding index of 2.66 ± 0.47 at baseline reduced to 0.38 ± 0.046 after one month. On comparison between the two groups after one month, experimental group showed a statistically significant difference (*p* = 0.021) (Table 3).

The present reduction in bleeding in the control group might be due to the resolution of gingival inflammation after scaling and root planning, while there was more reduction in the experimental group, which might be attributed to the fact that curcumin reduces inflammatory mediators and causes shrinkage by reducing inflammatory edema and vascular engorgement of connective tissues (Rastogi et al., 2012).

#### Plaque index (PI)

The plaque scores were measured using the Loe and Silness method, PI showed a statistically significant (*p* < 0.05) reduction after one month compared to baseline in control group as well as the experimental group (Table 3). This reduction in supragingival plaque score might be attributed to the chemical control of subgingival plaque by curcumin gel, which might also have an inhibitory effect on supragingival plaque. Moreover, good oral hygiene practiced by patients during the entire study period might have also increased the reduction in plaque. However, on comparison of mean plaque scores between both groups, statistically non-significant differences were recorded.

## Conclusion

In the current study curcumin, a well-established molecule in terms of safety and a multitude of medicinal activity have been well-formulated and characterized for periodontal application. *In-situ* gel formulations of curcumin were formulated with 30% pluronic F127 as a thermosensitive polymer and 1% carbopol P934 as a pH-sensitive and mucoadhesive polymer. The use of pluronic *in-situ* gel successfully produced stabilized curcumin formulation for periodontal application. *In-situ* gel formulation G_2_ containing 2% curcumin showed the most accepted results regarding gelation temperature, pH, ease of insertion into periodontal pocket, and controlled drug release for prolonged duration. Clinical studies on periodontitis have shown promising results regarding reduction of probing depth and bleeding index curcumin in this type of drug delivery system can serve as a novel approach for treating periodontal diseases with better patient compliance which truly hold a promising future in the therapeutic application of curcumin in dentistry.
